# Outcome of Hemiarthroplasty and Total Hip Replacement for Active Elderly Patients with Displaced Femoral Neck Fractures: A Meta-Analysis of 8 Randomized Clinical Trials

**DOI:** 10.1371/journal.pone.0098071

**Published:** 2014-05-22

**Authors:** Yiqiong Zhao, Dong Fu, Kai Chen, Guodong Li, Zhengdong Cai, Yan Shi, Xiaobing Yin

**Affiliations:** The Tenth People's Hospital Affiliated with Tong Ji University Shanghai, China; San Francisco Coordinating Center, United States of America

## Abstract

**Background:**

Displaced fracture of the femoral neck has been a common clinical problem, especially in aged patients. However, the optimal treatment choice remains controversial. The purpose of this study is to conduct a systematic review of randomized clinical trials assessing the results of hemiarthroplasty and total hip replacement in patients undergoing either alternative using meta-analysis.

**Methods:**

A literature search for randomized clinical trials was conducted through Medline, Embase and Cochrane library between 1969 and 2013 with no restrictions. Additional relevant articles were referred as source of information by way of manual searches on major orthopedic journals. Upon the search, two authors independently evaluated study quality and relevant data was extracted.

**Results:**

A total of 8 studies with 983 patients were included in this meta-analysis. After pooling the available data, a significant dominance of Harris hip score was found for total hip replacement compared with hemiarthroplasty (SMD: −7.11, 95%:−10.70,−3.53) one year postoperatively and the advantage kept over (SMD: −6.91, 95%:−12.98, −0.85) two years after surgery. A trend toward a higher dislocation rate was found in total hip replacement group (RR: 0.46, 95%: 0.21, 1.02), of which the difference was considered insignificant. The risk of revision in group hemiarthroplasty appeared to be more than two folds higher than that after total hip replacement (RR: 4.14, 95%CI: 2.09, 8.19).

**Conclusion:**

Even though there is a higher rate of dislocation after total hip replacement, this disadvantage could be accounted for, on the basis of a better functional score and the lower revision rate. However, from the results, it stands to reason that total hip replacement should be strongly suggested in elderly active patients with femoral neck fracture.

## Introduction

Displacement of femoral neck (fracture) in the elderly has become a worldwide health concern. The trend of rise in incidence with increasing age is alarming, and it is predicted that the total number of patients suffering femoral fracture would rise to 6.26 million per year by 2050 worldwide [1]. Treatment choices for femoral neck fractures in elderly patients include internal fixation, hemiarthroplasty (HA) and total hip replacement (THR). The option should be determined by the fracture typing, the patients' condition, functional demands and their medical and mental ability to cope with surgery [2], [3]. Currently, the optimal initial treatment of choice osteosynthesis which resulted in nonunion or avascular necrosis is fast given way to arthroplasty. It is recommended that Osteosynthesis should be favoured in young patients with displaced intracapsular fractures [4], relative to elderly patients who are medically fit for arthroplasty [5]. Arthroplasty is commonly used either as hemiarthroplasty or total hip arthroplasty. Although both two prosthetic replacement methods are widely accepted, the preferred treatment for displaced intracapsular fracture in elderly patients is currently of subject of debate among orthopedic surgeons. Theoretically, the results from previous prospective studies and meta-analysis [6], [7] which comparing the outcome of hemiarthroplasty and total hip replacement in patients with fracture of the femoral neck have equally been inconsistent. The purpose of this study is to evaluate the evidence from previous randomized clinical studies by summarizing it quantitatively with a meta-analysis approach.

## Methods

### Search Strategy

Computer literature search was conducted in Medline, Embase and Cochrane databases without special limitations, and articles ahead of publication were also included. The following Medical Subject Headings (MeSH) and terms were used in searching: “femoral neck fracture”, “hip fracture”, “arthroplasty”, “total hip replacement”, “prosthesis replacement” and “elderly”. Moreover, keywords in headers and abstracts in related journals were also used in the searches (e.g. Journal of Bone and Joint Surg). The reference list of each comparative study and previous reviews were hand searched to find additional studies. If the necessary data for analysis was absent or insufficient, we tried to contact the authors by e-mail. All processes in this review were in accordance with the standards of quality for meta-analysis [8].

### Eligibility Criteria

Citations selected from the primary search were screened for eligibility by two independent authors. We included the studies if they met all following criteria: 1 age >55 years; 2 displaced hip fracture (Garden III, IV); 3 mental health and independent walking prior to the fracture; 4 the intervention included hemiarthroplasty or total hip replacement regardless of whether internal fixation was compared in the trial; 5 pathologic and old fractures, patients with advanced rheumatoid arthritis or metastatic diseases were excluded. A controversy was cross-checked and resolved by a third author to reach a final consensus. This was done primarily to exclude experimental bias as much as possible thereby improving consistency.

### Data Extraction

Data were extracted independently by two authors subsequently after all the eligible studies were recruited. The following variables were recorded: last name of the first author, country where the study was performed, year of publication, participant sex and age, sample size, surgical approach. Outcome measurements including hip function (Harris hip score), revision (reasons) and dislocation rate and some parameters about the surgery (e.g. duration of surgery, blood loss, transfusion) were also recorded in detail.

### Statistical Analysis

Study-specific RR (relative risks) and associated 95% (CI) confidence intervals accounting for discontinuous variables within the study were pooled using a random-effects model, which considered both within-study and between-study variation. Standardized mean difference (SMD) or weighted mean difference (WMD) were used for continuous variables for which a fixed effect model was used initially, and if the P value of heterogeneity test was < 0.1 or I^2^>50%, the random effect model replaced the previous modality.

Statistical heterogeneity among studies was evaluated by both Q^2^ test [9] and I^2^ test [10]. Sensitivity was performed to evaluate the stability of the results. Subgroup analysis was conducted if the data was present.

An estimation of potential publication bias was assessed by the funnel plot, in which the SE (standardized effect) of log (RR) of each study was plotted against its log (RR). The result was assessed by the method of Egger's linear regression test, a linear regression approach to assess asymmetry on the natural logarithm scale of the RR. All statistical analysis were performed with STATA software (version 11.1; Stata Corporation, College Station, Texas) and Review Manager (version 5.0.0 for Windows, The Cochrane Collaboration, The Nordic Cochrane Centre, Copenhagen, 2008).

### Literature Search

**Figure 1:**
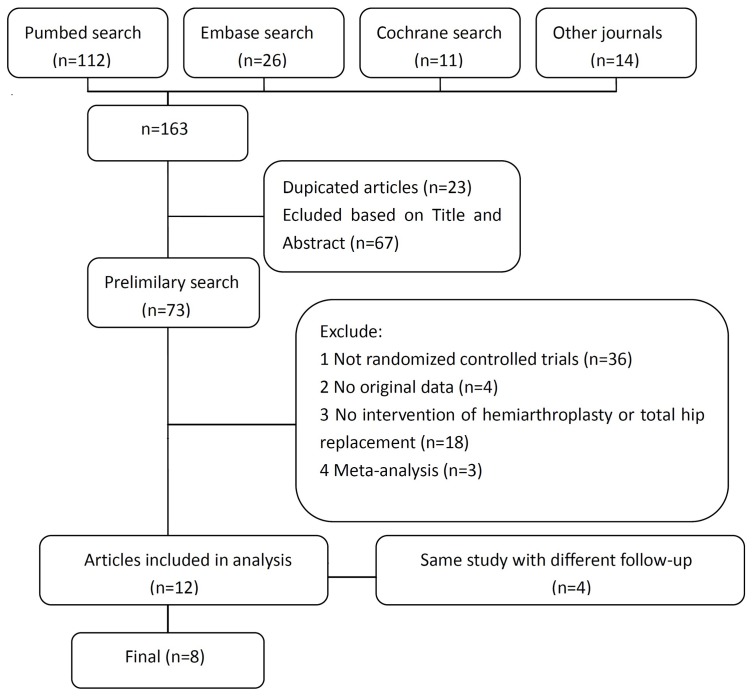
Flow chart of the studies recruited in this meta-analysis.

A flow chart of the studies recruited in our review was shown in Figure 1. We identified 163 potential citations (112 from Pubmed; 26 from Embase; 11 from the Cochrane Randomized Trials Databases; and 14 from relevant journals) aiming at comparing hemiarthroplasty and total replacement for the treatment of femoral neck fracture in elderly patients. After reading the articles, as well as communicating with the first author to get additional studies or data, 12 of the 163 citations were selected for application. Four studies belonging to the same institution were conducted with different follow-up and only the recent studies were selected. As a result, eight articles including a total of 983 patients (526 in the hemiarthroplasty group and 457 in the total hip replacement group) were used in the final analysis (details shown in Table 1).

**Table 1:** Summary of studies that compared groups of patients treated with HA and THA.

author	year	location	Male/Female	Surgical approach	Type	Age(year)	No(P)	Follow up (month)	Surgical Time(min)	Blood loss	Dislocation	revision
**Cadossi, et al**	2013	German	13/28	straight lateral	HA Bipolar	84.2	37	28.6	81	<500 ml 7/26#	NG	NG
			8/34		THA PCU	82.3	41	30.1	75.4	<500 ml 15/27	NG	NG
**Giannini, et al**	2011	Italy	NG	NG	HA Bipolar	84.2	26	12	71.6	380	NG	NG
					THA PCU	82.3	26	12	76.7	460	NG	NG
**Hedbeck, et al**	2011	Sweden	6/54	anterolateral	HA Bipolar	80.7±5.1	60	48	NG	NG	0	0 (HA)
			13/47		THR	80.5±5.1	60	48	NG	NG	0	1 (THA) for infection
**Avery, et al**	2011	UK	9/32	transgluteal lateral	HA Unipolar	75.83	41	103	NG	NG	0	1 for erosion 3 for peri-prosthetic fracture(HA)
			8/32		THA PCA	74.2	40	106	NG	NG	3	1 for femoral stem subsidence (THA)
**Vanden, et al**	2010	Holland	22/115	anterolateral posterolateral	HA Bipolar	80.3	137	60	<90 min 110	<500 ml 111	0	5 for erosion 1 for acetabulum osteoarthritis (HA)
			25/90		THA	82.1	115	60	<90 min 75	<500 ml 70	8	1 for acetabulum osteoarthritis 1 for infection(THA)
**Macaulay, et al**	2008	USA	9/14	Posterolateral direct lateral	Uni(Bi)polar	77±9	23	12	82.0±35.1	5.4±2.8(Unit)	0	0 (HA)
			10/7		THA	82±7	17	12	89.1±35.8	7.7±5.5(Unit)	1	1 for dislocation(THA)
**Keating, et al**	2006	UK	57/54	lateral posterior	Uni(Bi)polar	75.4±7	111	24	58.5	NG	2/69#	NG
			17/52		THA	75.2±6	69	24	79.7	NG	3/69	NG
**Kasetti, et al**	2000	UK	NG	posterolateral	HA Bipolar	82.06	91	156	NG	NG	12	19 for acetabular erosion, loosening, heterotopic ossification 3 for deep infection)(HA)
					THA	81.03	89	156	NG	NG	18	2 for infection; 4 for dislocation)(THA)

Abbreviations: NG = not given; HA = hemoarthroplasty; THA = total hip arthroplasty; PCU = acetabular polycarbonate-urethane cup; PCA = polyethylene cemented acetabular;

No(p) = number of patients;

# means patients available for the clinical data.

### Study Characteristics

The eight studies on arthroplasty (hemiarthroplasty and total hip replacement) were published between 2000 and 2013 (Table 1). Out of the eight studies, three were conducted in the United Kingdom, the other five trials were completed in USA, Holland, Germany, Italy and Sweden respectively. At baseline, there were no significant differences between the groups of hemiarthroplasty and total hip replacement.

All eight trials have adequate sequence generation for randomization. Allocation concealment by either a sealed-envelope technique [11], [12] or computer automation based distribution [13], [14] was performed in four studies while the remained four were unclear [15]–[18]. In terms of blinding, it was “unclear” for four trials, “no” for two [11], [15] and “yes” for two [14], [16]. All the randomized trials were free of selective reporting. Whether these eight trials were free of other elements of bias could not be found.

## Results

### Harris hip score

HSS at 3 m, 6 m, 1 y, 2 y, 3 y and 5 y postoperatively were recorded and comparison between group HA and THR based on the available score 1 y and 2 y after surgery was performed. A greater likelihood of higher Harris hip score was observed in the total hip replacement group compared to the hemiarthroplasty group (SMD: −7.11, 95%:−10.70,−3.53). This dominance kept over time (SMD: −6.91, 95%:−12.98, −0.85) two years after surgery. Both results demonstrated significant differences.

### Dislocation rate

**Figure 2:**
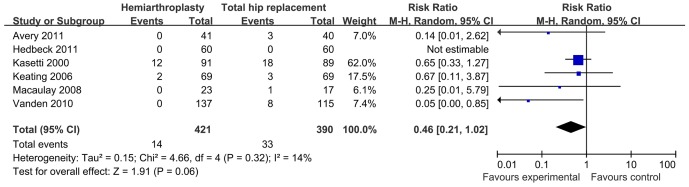
Statistical analysis of dislocation between HA (hemiarthroplasty) and THA (total hip arthroplasty) with Review Manager.

Six studies provided information about dislocation. The Review Manage Software will exclude the categorical variables automatically if the value of incidence is 0% in both groups. Except one study in which no dislocation occurred in either group, a total of 5 studies with 691 patients were included for analysis. A trend toward a higher dislocation rate was found in total hip replacement group (RR: 0.46, 95%: 0.21, 1.02) with a low heterogeneity (I^2^ = 14%, p = 0.32) Figure 2.

### Revision rate

**Figure 3:**
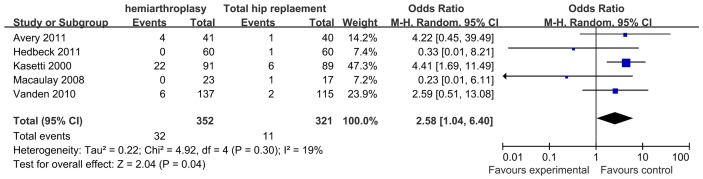
Statistical analysis of dislocation between HA (hemiarthroplasty) and THA (total hip arthroplasty) with Review Manager.

Based on the five studies providing available data, the risk of revision in group hemiarthroplasty appeared to be more than two folds higher than that after total hip replacement (RR: 4.14, 95%CI: 2.09, 8.19)with a low heterogeneity (I^2^ = 19%, p = 0.30) Figure 3. Reasons for revision surgery in HA group were erosion 78.1% (25/32), peri-prosthetic fracture 9.4% (3/32), acetabulum osteoarthritis 3.1% (1/32) and deep infection 9.4% (3/32). While in THA group, infection, femoral stem subsidence, acetabulum osteoarthritis and dislocation occupied 36.4% (4/11), 9.1% (1/11), 9.1% (1/11) and 45.5% (5/11) respectively.

## Discussion

The goal of any surgical treatment for elderly and independent patients with displaced fractures of the femoral neck is to return them as quickly as possible to pre-injure functional status as well as minimize the risk of dislocation or reoperation. Total hip replacement and hemiarthroplasty are two primary treatment choices for those patients and each of these methods has its associated advantages and disadvantages.

Two meta-analysis [6], [7] evaluated the optimal type of arthroplasty before this study was initiated. Both studies concluded that total hip replacement was associated with a better overall result compared to hemiarthroplasty in the management of elderly patients, but it remained to be further confirmed, due to insufficient evidence backing the claim. An obvious limitation of their studies was that the conclusion was based on relatively small studies. This current meta-analysis summarized the results of randomized controlled trials, including eight randomized clinical studies on hemiarthroplasty and total hip replacement with a total of 983 patients. This meta-analysis up to now is associated with the largest number of patients. The results indicated that total hip replacement had a potential advantage on the whole, compared to hemiarthroplasty.

### Harris hip score

As one of the most important measurement tools, Harris hip score was used to investigate the living status of patients. From three independent studies [14], [18], [19], we extracted that hip function reached its peak one year after surgery. Our result favored the total hip replacement because hip function improved significantly compared to the hemiarthoplasty group within 1year and 2year after surgery. Cadossi et al noted from 3 m to 3 y postoperatively, the higher HHS transferred from HA to THR and the dominance of THR seemed to be increasingly evident. Similar result was reported by Hedbeck with the same duration of follow-up (Table 2). Avery et al [17] showed the functional score had declined in both groups between three and nine years and the dominance in the THR group had also decreased nine years after surgery as a result of the older age, prosthetic degeneration and other complications. Hemiarthroplasty was often accompanied with acetabular erosion and protrusion particularly in young patients under 60 years [17], [20]. According to our analysis, erosion accounted for 78.1% revision surgery of patients in HA group while no erosion case was found in THP group. Theoretically, acetabular erosion was associated with hip pain and poor function. This phenomenon would be increasingly apparent with the longer follow-up.

**Table 2:** Summary of HSS of included five studies that compared groups of patients treated with HA and THA.

author	Type	No (P)	HSS					
			3month	6month	1year	2year	3year	5year
**Cadossi, et al**	HA	37	74.0 (43.7 to 93)	NG	73.1 (40 to 97.4)	71.9 (30 to 93)	71.3 (45 to 93)	NG
	THR	41	72.3 (50.3 to 98)	NG	74.7 (56 to 98)	75.0 (55 to 98)	78.7 (55 to 98)	NG
**Giannini, et al**	HA	26	71.6	NG	75.5	NG	NG	NG
	THR	26	74.	NG	80.7	NG	NG	NG
**Hedbeck, et al**	HA	60	NG	NG	79.4±12.3	77.9±12.5	75.2±15.4	NG
	THR	60	NG	NG	87.2±9.4	87.2±10.1	89.0±8.1	NG
**Vanden, et al**	HA	137	NG	NG	73.9 (23 to 100)	NG	NG	71.9 (33 to 99)
	THR	115	NG	NG	76.0 (44 to 100)	NG	NG	75.2 (45 to 96)
**Macaulay, et al**	HA	23	NG	77.1±12	80.6±14.3	81.1 ±11.7	NG	NG
	THR	17	NG	76.1±26.5	84.2±12.0	84.0±12.2	NG	NG

Abbreviations: No(P)  =  number of knees(patients); HSS  =  Harris hip score; NG  =  not given; HA = Hemiarthroplasty; THA: Total Hip Arthroplasty.

### Dislocation rate

Dislocation of the prosthesis has been the major concern after a primary total hip replacement [19], [21]. We tried to find a relationship between surgical approach and dislocation. However, it was impossible as mixed surgical approaches were used in each group. A higher rate of dislocation was reported previously in total hip replacement [16], [18]. In patients using a posterolateral approach during the surgery, reported incidence of dislocation ranged between 13% to 22% [18], [22]–[24]. Potential risk factors leading to dislocation include component design, operative approach, and around soft-tissue tensioning [25]. In our series, the modular femoral component with a 28 mm head was used in three studies, a 32 mm head in two studies and the remained were unknown. Dislocation rates for all approaches decrease as femoral head size increases from 22 mm to 32 mm [19]. However, of these five studies, no significant difference was observed between two different sizes of the femoral head and dislocation incidence. The reason which might explain this fact is that size 28 mm and 32 mm are two most commonly used head and any more increase in head size will produce a higher wear rate and subsequent aseptic loosening rate [26]. Surgical approach has been an important factor that influences the stability of hip joint. Woo and Morrey et al [25] reported a much higher dislocation rate for the posterior approach (5.8%) than that for anterolateral approach (2.3%) (p<0.01). In this review, six studies provided the data about dislocation. Hedback noticed no occurrence of dislocation in patients with anterolateral approach in either group, thus confirming that dislocation is not a major problem when an anterolateral approach is used [14], [21], [27], [28]. When the posterolateral approach was used, the highest dislocation rate in both groups among the six trials was observed (Table 1). However, the dislocation rate of THR with a posterior soft tissue repair was significantly lower than that without a posterior soft tissue repair in the posterolateral approach [29]. Consequently, if posterior structures could not be preserved and repaired as much as possible, anterolateral approach might be chosen for major patients just as recommended by Enocson [27].

### Erosion and Revision

Acetabular erosion is a very severe complication of both unipolar and bipolar HA (reaching to 36% and 26% of hips, respectively) after five years of follow-up postoperatively which often leading to revision surgery [30]–[32]. In younger patients, this change would be more frequent because of more activity. Acetabular erosion has been considered to be one important factor influencing the functional outcome. The majority of the patients receiving hemiarthroplasty experienced revision surgery which might result from the degeneration of acetabular cartilage or erosion of the prosthesis [16], [30]. In the hemiarthroplasty group, the bipolar prosthesis usually had a better result than the unipolar prosthesis [15]. In this study, a bipolar component was used in 90% of the patients undergoing hemiarthroplasty and a monopolar component for the other 10% of the patients. Even though, the revision rate of HA group appeared to be more than two folds higher than that after THR, 78% resulted from erosion in HA group. When we linked the data from 12 month to 156 month of five inclusive studies, another interesting and obvious finding was that compared to HA, THR revealed notable dominance of relatively lower increasing rate of revision with the passage of time. Patients who had a ‘secondary’ total hip replacement for a failed hemiarthroplasty would feel much better than those who retained their hemiarthroplasty (e.g less pain and better functional outcome). But they were less likely to restore their independency than those who undertook the same procedure initially [17]. Possible reasons might be the experience of suffering two surgeries at different periods and more complications after the secondary surgery. To reduce the rate of erosion, revision and subsequent bad functional results, THR should replace HA from the beginning in some patients.

### Other measurements

#### Blood loss and Transfusion

Patients who underwent THR had a slightly higher blood loss than those in HA group (460 vs 320 ml) in the study of Giannini et al. Hedbeck et al reported a smaller proportion of patients who had blood loss over 500 ml in the hemiarthroplasty group (64.3% vs. 93.3%). Macaulay et al noted that more blood transfusion was needed in THR compared to HA (7.7 vs 5.5 unit). However, Cadossi et al recently got an opposite result that more patients undertaking THR had blood loss of less than 500 ml. Total to say, no advantage in either group was observed in this review and more studies providing unified, concrete values are needed.

#### Surgical time

The mean duration of surgery was a litter longer in THR. Vander et al divided surgical time into three periods and the results were less than one hour (35.2% vs. 9.5%), one to one and a half hour (52.8% vs.61.9%), more than one and a half hour (12% vs. 28.6%) in the HA and THR group respectively. Keating, Macaulay, Giannini observed longer mean surgical time in THR group, while Cadossi found a decrease of 5.6 minutes in THR group. An important possible factor explaining this difference was that with the development of technique and prosthesis, the surgery of THR tended to be more convenient and time saving. Of course, individual differences could not be ignored too.

However, we failed to calculate whether there was a significant difference between these two groups regarding blood loss and surgical time as a result of various value forms. (Table 1)

## Strengths and Limitations of This Analysis

Meta-analysis is an effective tool for revealing trends that might not be apparent in a single study. Pooling the data from different high quality studies increases the confidence of the findings regarding the parameters which were addressed in the included studies. The current analysis met the methodological standard with no or low heterogeneity. The number of total patients were large enough and all participants met the including criteria. All of our evaluations were based on randomized controlled trials, which minimize the possibility of recall or selection bias.

To our knowledge, this review is the most current report on this topic. Here, we only included RCTs, the patients were balanced distribution between the two groups. This enhanced the credibility of our statistical result. Despite these advantages, the limitations should be acknowledged separately. Firstly, necessary data were not provided in each trial which may lead to less accuracy of the final conclusion. Secondly, because the number of studies involved in this analysis was relatively small, some of the subgroup analyses were not performed (e.g. age, sex and country). Another important limitation for generalizing our results was that we could not assessed the health-related quality according to some index scores like EQ-5D because of limited data. So the results should be verified by more centers with a longer follow-up in further trials.

## Conclusion

An obvious advantage was found in Harris hip score, revision ratewith significant difference for the total hip replacement. Even though dislocation rate was a little higher in THR, it could be treated with manipulative reduction in most cases. Regarding all these results, it could be concluded that total hip replacement should be the first choice of treatment in elderly and independent patients with fracture of the femoral neck.

## Supporting Information

Checklist S1 **PRISMA Checklist.**
(DOC)

Diagram S1 **PRISMA 2009 Flow Diagram.**
(DOC)

## References

[1] Riggs BL, Melton LJ III (1995) The worldwide problem of osteoporosis: insights afforded by epidemiology. Bone 17: 505s–511s. 8573428 10.1016/8756-3282(95)00258-4

[2] Parker MJ, Gurusamy K (2006) Internal fixation versus arthroplasty for intracapsular proximal femoral fractures in adults. Cochrane Database Syst Rev: Cd001708. 10.1002/14651858.CD001708.pub2 PMC8407320 17054139

[3] Parker MJ, Gurusamy KS, Azegami S (2010) Arthroplasties (with and without bone cement) for proximal femoral fractures in adults. Cochrane Database Syst Rev: Cd001706. 10.1002/14651858.CD001706.pub4 PMC12360749 20556753

[4] Bhandari M, Devereaux PJ, Swiontkowski MF, Tornetta P III, Obremskey W, et al (2003) Internal fixation compared with arthroplasty for displaced fractures of the femoral neck. A meta-analysis. J Bone Joint Surg Am 85-a: 1673–1681. 12954824 10.2106/00004623-200309000-00004

[5] Parker MJ, Khan RJ, Crawford J, Pryor GA (2002) Hemiarthroplasty versus internal fixation for displaced intracapsular hip fractures in the elderly. A randomised trial of 455 patients. J Bone Joint Surg Br 84: 1150–1155. 12463661 10.1302/0301-620x.84b8.13522

[6] Goh SK, Samuel M, Su DH, Chan ES, Yeo SJ (2009) Meta-analysis comparing total hip arthroplasty with hemiarthroplasty in the treatment of displaced neck of femur fracture. J Arthroplasty 24: 400–406. 18701252 10.1016/j.arth.2007.12.009

[7] Liao L, Zhao J, Su W, Ding X, Chen L, et al (2012) A meta-analysis of total hip arthroplasty and hemiarthroplasty outcomes for displaced femoral neck fractures. Arch Orthop Trauma Surg 132: 1021–1029. 22446696 10.1007/s00402-012-1485-8

[8] Stroup DF, Berlin JA, Morton SC, Olkin I, Williamson GD, et al (2000) Meta-analysis of observational studies in epidemiology: a proposal for reporting. Meta-analysis Of Observational Studies in Epidemiology (MOOSE) group. Jama 283: 2008–2012. 10789670 10.1001/jama.283.15.2008

[9] Wu R, Li B (1999) A multiplicative-epistatic model for analyzing interspecific differences in outcrossing species. Biometrics 55: 355–365. 11318188 10.1111/j.0006-341x.1999.00355.x

[10] Higgins JP, Thompson SG, Deeks JJ, Altman DG (2003) Measuring inconsistency in meta-analyses. Bmj 327: 557–560. 12958120 10.1136/bmj.327.7414.557 PMC192859

[11] Cadossi M, Chiarello E, Savarino L, Tedesco G, Baldini N, et al (2013) A comparison of hemiarthroplasty with a novel polycarbonate-urethane acetabular component for displaced intracapsular fractures of the femoral neck: a randomised controlled trial in elderly patients. Bone Joint J 95-b: 609–615. 23632669 10.1302/0301-620X.95B5.31083

[12] Macaulay W, Nellans KW, Iorio R, Garvin KL, Healy WL, et al (2008) Total hip arthroplasty is less painful at 12 months compared with hemiarthroplasty in treatment of displaced femoral neck fracture. Hss j 4: 48–54. 18751862 10.1007/s11420-007-9061-4 PMC2504272

[13] van den Bekerom MP, Hilverdink EF, Sierevelt IN, Reuling EM, Schnater JM, et al (2010) A comparison of hemiarthroplasty with total hip replacement for displaced intracapsular fracture of the femoral neck: a randomised controlled multicentre trial in patients aged 70 years and over. J Bone Joint Surg Br 92: 1422–1428. 20884982 10.1302/0301-620X.92B10.24899

[14] Keating JF, Grant A, Masson M, Scott NW, Forbes JF (2006) Randomized comparison of reduction and fixation, bipolar hemiarthroplasty, and total hip arthroplasty. Treatment of displaced intracapsular hip fractures in healthy older patients. J Bone Joint Surg Am 88: 249–260. 16452734 10.2106/JBJS.E.00215

[15] Giannini S, Chiarello E, Cadossi M, Luciani D, Tedesco G (2011) Prosthetic surgery in fragility osteopathy. Aging Clin Exp Res 23: 40–42. 21970918

[16] Hedbeck CJ, Enocson A, Lapidus G, Blomfeldt R, Tornkvist H, et al (2011) Comparison of bipolar hemiarthroplasty with total hip arthroplasty for displaced femoral neck fractures: a concise four-year follow-up of a randomized trial. J Bone Joint Surg Am 93: 445–450. 21368076 10.2106/JBJS.J.00474

[17] Avery PP, Baker RP, Walton MJ, Rooker JC, Squires B, et al (2011) Total hip replacement and hemiarthroplasty in mobile, independent patients with a displaced intracapsular fracture of the femoral neck: a seven- to ten-year follow-up report of a prospective randomised controlled trial. J Bone Joint Surg Br 93: 1045–1048. 21768626 10.1302/0301-620X.93B8.27132

[18] Ravikumar KJ, Marsh G (2000) Internal fixation versus hemiarthroplasty versus total hip arthroplasty for displaced subcapital fractures of femur—13 year results of a prospective randomised study. Injury 31: 793–797. 11154750 10.1016/s0020-1383(00)00125-x

[19] Berry DJ, von Knoch M, Schleck CD, Harmsen WS (2005) Effect of femoral head diameter and operative approach on risk of dislocation after primary total hip arthroplasty. J Bone Joint Surg Am 87: 2456–2463. 16264121 10.2106/JBJS.D.02860

[20] Soreide O, Skjaerven R, Alho A (1982) The risk of acetabular protrusion following prosthetic replacement of the femoral head. Acta Orthop Scand 53: 791–794. 7136591 10.3109/17453678208992294

[21] Tidermark J, Ponzer S, Svensson O, Soderqvist A, Tornkvist H (2003) Internal fixation compared with total hip replacement for displaced femoral neck fractures in the elderly. A randomised, controlled trial. J Bone Joint Surg Br 85: 380–388. 12729114 10.1302/0301-620x.85b3.13609

[22] Waaler Bjornelv GM, Frihagen F, Madsen JE, Nordsletten L, Aas E (2012) Hemiarthroplasty compared to internal fixation with percutaneous cannulated screws as treatment of displaced femoral neck fractures in the elderly: cost-utility analysis performed alongside a randomized, controlled trial. Osteoporos Int 23: 1711–1719. 21997224 10.1007/s00198-011-1772-1

[23] Johansson T, Jacobsson SA, Ivarsson I, Knutsson A, Wahlstrom O (2000) Internal fixation versus total hip arthroplasty in the treatment of displaced femoral neck fractures: a prospective randomized study of 100 hips. Acta Orthop Scand 71: 597–602. 11145387 10.1080/000164700317362235

[24] Jonsson B, Sernbo I, Carlsson A, Fredin H, Johnell O (1996) Social function after cervical hip fracture. A comparison of hook-pins and total hip replacement in 47 patients. Acta Orthop Scand 67: 431–434. 8948244 10.3109/17453679608996662

[25] Woo RY, Morrey BF (1982) Dislocations after total hip arthroplasty. J Bone Joint Surg Am 64: 1295–1306. 7142237

[26] Halley D, Glassman A, Crowninshield RD (2004) Recurrent dislocation after revision total hip replacement with a large prosthetic femoral head. A case report. J Bone Joint Surg Am 86-a: 827–830. 15069152 10.2106/00004623-200404000-00025

[27] Enocson A, Hedbeck CJ, Tidermark J, Pettersson H, Ponzer S, et al (2009) Dislocation of total hip replacement in patients with fractures of the femoral neck. Acta Orthop 80: 184–189. 19404800 10.3109/17453670902930024 PMC2823165

[28] Enocson A, Tidermark J, Tornkvist H, Lapidus LJ (2008) Dislocation of hemiarthroplasty after femoral neck fracture: better outcome after the anterolateral approach in a prospective cohort study on 739 consecutive hips. Acta Orthop 79: 211–217. 18484246 10.1080/17453670710014996

[29] Suh KT, Park BG, Choi YJ (2004) A posterior approach to primary total hip arthroplasty with soft tissue repair. Clin Orthop Relat Res: 162–167. 10.1097/00003086-200401000-00026 15043109

[30] Rodriguez-Merchan EC (2002) Displaced intracapsular hip fractures: hemiarthroplasty or total arthroplasty? Clin Orthop Relat Res: 72–77. 12011696

[31] Baker RP, Squires B, Gargan MF, Bannister GC (2006) Total hip arthroplasty and hemiarthroplasty in mobile, independent patients with a displaced intracapsular fracture of the femoral neck. A randomized, controlled trial. J Bone Joint Surg Am 88: 2583–2589. 17142407 10.2106/JBJS.E.01373

[32] Poignard A, Bouhou M, Pidet O, Flouzat-Lachaniette CH, Hernigou P (2011) High dislocation cumulative risk in THA versus hemiarthroplasty for fractures. Clin Orthop Relat Res 469: 3148–3153. 21773860 10.1007/s11999-011-1987-7 PMC3183183

