# Supplementary material for: Outcome of Hemiarthroplasty and Total Hip Replacement for Active Elderly Patients with Displaced Femoral Neck Fractures: A Meta-Analysis of 8 Randomized Clinical Trials
Source: PLoS One. 2014 May 22;9(5):e98071. doi: 10.1371/journal.pone.0098071 (PMC4031167; doi:10.1371/journal.pone.0098071)
Supplement: Diagram S1 — PRISMA 2009 Flow Diagram. (DOC) [file pone.0098071.s002.doc]

**Flow Diagram of the studies recruited in this meta-analysis**

**Screening**

**Included**

**Eligibility**

**Identification**

Records identified through database searching
(n =149 )

Additional records identified through other sources
(n = 14 )

Records after duplicates removed
(n =163 )

Records screened
(n =140 )

Records excluded
(n = 23 )

Full-text articles assessed for eligibility
(n =73 )

Full-text articles excluded, with reasons
(n = 67 )

Studies included in qualitative synthesis
(n = 12 )

Studies included in quantitative synthesis (meta-analysis)
(n =8 )
